# Eicosapentaenoic acid restores inflammation-induced changes in chondrocyte mechanics by suppressing the NF-κB p65/CD44 signaling pathway and attenuates osteoarthritis

**DOI:** 10.1038/s12276-025-01529-7

**Published:** 2025-09-01

**Authors:** Qihao Yang, Jianqun Wu, Songqiang Huang, Jing Zeng, Yihan Jing, Zhiyong Wang, Chi Zhang, Zenghui Wu, Song Liu, Zhao Wang

**Affiliations:** 1 Department of Orthopaedic Surgery, Guangzhou Key laboratory of Spine Disease Prevention and Treatment, Guangdong Provincial Key Laboratory of Major Obstetric Diseases, Guangdong Provincial Clinical Research Center for Obstetrics and Gynecology, The Third Affiliated Hospital, Guangzhou Medical University, Guangzhou, China; 2 Department of Pain, Hubei No. 3 People’s Hospital of Jianghan University, Wuhan, China; 3 Department of Traumatic Surgery, Center for Orthopaedic Surgery, The Third Affiliated Hospital of Southern Medical University, Guangzhou, China; 4 School of Biomedical Sciences, Hunan University, Changsha, China; 5 Department of Orthopedics, Liwan Central Hospital of Guangzhou, Guangzhou, China

**Keywords:** Actin, Mechanisms of disease, Osteoarthritis

## Abstract

Here we investigate the effects and mechanisms of eicosapentaenoic acid (EPA) in regulating chondrocyte mechanics during inflammation in the progression of osteoarthritis (OA). Primary porcine chondrocytes and human OA chondrocytes were isolated and cultured. Cell mechanical properties were measured using atomic force microscopy. RNA sequencing, immunocytochemistry, quantitative PCR and western blotting were used to elucidate associated signaling mechanisms. Porcine cartilage and human OA cartilage explants were collected. Human articular cartilage samples were obtained from ten donors. Anterior cruciate ligament transection surgery was performed to induce OA in male C57BL/6J mice. The therapeutic effects of EPA and activation of associated signaling were evaluated using histology, immunohistochemistry and micro-computed tomography. EPA reduced F-actin intensity and Young’s modulus in IL-1α-treated porcine chondrocytes and in human OA chondrocytes. Mechanistically, EPA inhibited IL-1α-induced increase in CD44 expression in porcine chondrocytes by suppressing phosphorylation of NF-κB subunits p65. In addition, EPA alleviated articular cartilage degeneration and decreased the expression of p-p65 and CD44 in IL-1α-treated porcine and human OA cartilage explants. Moreover, EPA suppressed the increase in Young’s modulus induced by CD44 ligands (A6 peptide and low-molecular-weight hyaluronic acid) in porcine chondrocytes. Finally, intraarticular injection of EPA emulsion-integrated hyaluronic acid injection attenuated OA-associated alterations in articular cartilage and subchondral bone and decreased CD44 expression in mice. Our data not only provide new insights into EPA’s chondroprotective actions and underlying mechanisms but also offer new evidence supporting the therapeutic efficacy of a novel EPA emulsion-integrated hyaluronic acid injection for OA treatment.

## Eicosapentaenoic acid restores chondrocyte mechanics in osteoarthritis

Osteoarthritis (OA) is a common joint disease that causes pain and disability. It involves complex factors such as inflammation and mechanical stress, yet no effective drugs currently exist. This study explores how eicosapentaenoic acid (EPA), an omega-3 fatty acid, affects OA. Researchers used cells from pigs and humans to see how EPA influences chondrocyte mechanics. They treated these cells with EPA and an inflammatory substance called IL-1α to mimic OA conditions. They found that EPA helps maintain the cells’ normal shape and stiffness and reduces harmful proteins. EPA also lowers the expression of CD44, a protein linked to OA progression, by blocking a specific signaling pathway (NF-κB). In animal models, EPA reduced cartilage damage and improved joint health. The study suggests that EPA could be a promising treatment for OA by targeting inflammation and mechanical stress in joints.

This summary was initially drafted using artificial intelligence, then revised and fact-checked by the author.

## Introduction

Osteoarthritis (OA) is the most common form of arthritis and was ranked as the 18th leading cause of disability-adjusted life years in the 50–74 age group, according to the Global Burden of Disease 2019 study^1^. The etiology of OA is multifactorial, including mechanical overloading, inflammation, aging, obesity, gender, genetics and trauma^2,3^. The combination of various risk factors leads to a complex pathogenesis in patients with OA characterized by the structural destruction of the entire joint. So far, no disease-modifying OA drugs have been approved, largely due to our insufficient understanding of the pathological mechanisms involved in OA development.

Although the exact trigger of OA remains unknown, the disease progression is closely associated with the degradation of the cartilage extracellular matrix (ECM) and involves complex interactions between mechanical and inflammatory factors (for review, see ref.^4^). Decades of research have demonstrated that chondrocytes sense and respond to abnormal biomechanical changes through mechanosensing mechanisms, leading to cytoskeletal reorganization, activation of inflammatory signaling, loss of chondrocyte phenotype and degradation of the cartilage ECM. In the future, developing disease-modifying OA drugs and therapeutics that target mechanosignals to disrupt the vicious cycle between pathological mechanical and inflammatory processes may offer a promising solution for this complex disease.

Omega-3 polyunsaturated fatty acid (PUFA), including eicosapentaenoic acid (EPA), docosahexaenoic acid (DHA) and alpha-linolenic acid (ALA), are known for their anti-inflammatory effects in OA, whereas omega-6 PUFAs tend to promote inflammation. A comparative study demonstrated that EPA was the most effective omega-3 PUFA at suppressing mRNA levels of key OA-related proteins, followed by DHA and ALA^5^. Indeed, extensive preclinical and clinical studies have investigated the effects of EPA in OA. In vitro studies have shown that EPA can reduce the expression of various pro-inflammatory mediators, ECM catabolic enzymes, cyclooxygenase-2 (COX-2) and inducible nitric oxide synthase, thereby attenuating inflammatory response, ECM degradation, prostaglandin E2 (PGE2) production and apoptosis in isolated chondrocytes^5–12^. In vivo studies have demonstrated that altering circulating serum levels of omega-3 PUFAs can alleviate obesity-associated OA^13^, posttraumatic OA^14–18^ and hip/stifle OA^19–22^ in various animal models. In addition, human studies investigating the effects of dietary or supplemental interventions containing omega-3 PUFAs in OA management have reported notable improvements in pain relief and joint function^23–27^. In summary, previous studies provide credible evidence for the use of EPA as a potential therapeutic option in OA, primarily due to its anti-inflammatory properties. However, the role of EPA in chondrocyte mechanics during OA pathogenesis remains unclear, and their mechanistic association has yet to be elucidated.

Interleukin-1 (IL-1), a key pro-inflammatory mediator, plays a crucial role in OA pathogenesis and has been widely used in in vitro OA models to investigate the influence of mechanical and inflammatory factors on disease progression. Recent studies have shown that IL-1 regulates multiple mechanosignals in chondrocytes, including Piezo1, primary cilia, integrins and CD44, thus causing associated changes in F-actin organization and cell mechanical properties. IL-1α upregulated Piezo1 expression, thereby increasing intracellular Ca^2+^ concentration and inducing F-actin rarefaction via the transcription factors (TFs) CREB1, ATF2 and HNF4α in porcine chondrocytes^28^. IL-1β increased primary cilia length and associated inflammatory response through a protein kinase A (PKA)-dependent mechanism in bovine chondrocyte^29^. Loss of integrin α1β1 reduced the intracellular calcium transient response to IL-1α in chondrocytes isolated from integrin α1-null mice^30^. CD44 is a nonkinase transmembrane glycoprotein ubiquitously expressed in a variety of cell types. In cancer cells, binding of CD44 to its ligands in the ECM activates various downstream signaling pathways that regulate cytoskeleton rearrangement, cell adhesion, differentiation, proliferation and survival (for review, see ref. ^31^). Studies from Knudson’s group have shown that IL-1α stimulates CD44 expression in normal human articular cartilage slices, isolated human chondrocytes and porcine chondrocytes^32,33^. However, the effects of EPA on IL-1-induced changes in mechanosignaling pathways in chondrocytes have not been investigated.

This study aims to address the unanswered question of whether EPA plays a role in regulating chondrocyte mechanics during OA pathogenesis. We assessed F-actin organization and cell mechanical properties in IL-1α-treated porcine chondrocytes and human OA chondrocytes. mRNA sequencing data from porcine chondrocytes, combined with a human gene expression profile dataset from the Gene Expression Omnibus, were used to identify differentially expressed genes (DEGs). The therapeutic effects of EPA and activation of associated signaling pathway were further confirmed in IL-1α-treated porcine cartilage explants, human OA cartilage explants and a mouse OA model.

## Materials and methods

### Isolation of primary porcine chondrocytes and human OA chondrocytes

Porcine chondrocytes were isolated from full-depth cartilage obtained from porcine metacarpophalangeal joints through a process of sequential enzyme digestion using pronase and collagenase as previously described^34^. Isolated porcine chondrocytes were cultured in Dulbecco’s modified Eagle medium (DMEM, Gibco, 11054020) supplemented with 15% fetal bovine serum (Gibco, 10099141), 1% penicillin–streptomycin (Gibco, 15140122), 10 mM HEPES (Sigma, H0887), 4 mM l-glutamine (Corning, 25005CI) and 0.15 mg/ml l-ascorbic acid (Sigma, A4544).

Human articular cartilage samples were obtained from three male and seven female donors (age 60–81 years, mean 70 years) who underwent total knee arthroplasty after obtaining written informed consent, as approved by the Institutional Review Board of the Third Affiliated Hospital of Guangzhou Medical University (no. 2023-075). Human OA chondrocytes were isolated through enzymatic digestion in a manner similar to porcine chondrocytes. Isolated human chondrocytes were cultured in DMEM supplemented with 10% fetal bovine serum and 1% penicillin–streptomycin. Cells were seeded and cultured at 37 °C with 5% CO_2_ for 24 h without passaging, and then subjected to the following experiments.

### Chondrocyte treatment with EPA and IL-1α

EPA (Sigma, E2011) was prepared in a complex with defatted bovine serum albumin (BSA, Sigma, A8806) as previously described^12^. In brief, 300 µg/ml EPA–BSA complex (20 mM HEPES, 140 mM NaCl, 4.5 mM KCl, 1 mM MgCl_2_, 2.5 mM CaCl_2_, 11 mM glucose and 3.5 mg BSA, pH 7.4) was incubated at 37 °C for 16 h. EPA–BSA complex was then added to cells to achieve a final EPA–BSA concentrations of 10, 30, 50, 100 and 200 µg/ml. Porcine chondrocytes were treated with EPA for 8 h and then stimulated with 10 ng/ml IL-1α (PeproTech, 20001A) for 24 h, while human OA chondrocytes were treated with EPA for 8 h.

### Cell viability assay

Cell viability was assessed using Cell Counting Kit-8 (CCK-8, GlpBio, GK10001). Freshly isolated porcine chondrocytes were seeded in a 96-well plate at a density of 20,000 cells per well in 200 μl of culture medium. Then, chondrocytes were cultured overnight and treated with or without various concentrations of EPA (10, 30, 50, 100 and 200 µg/ml) for 48 h. Chondrocytes were then treated with 20 μl of CCK-8 solution for 4 h at 37 °C in an incubator. Absorbance was measured at 450 nm using a Bio-Rad Microplate Reader.

### TUNEL assay

DNA fragmentation, a hallmark of late apoptosis, was detected using the terminal deoxynucleotidyl transferase dUTP nick-end labeling (TUNEL) assay (Beyotime, C1086). In brief, after EPA treatment, porcine chondrocytes cultured on coverslips were fixed with 4% paraformaldehyde for 15 min at room temperature, followed by permeabilization with 0.1% Triton X-100 for 10 min. Cells were incubated with TUNEL reaction mixture for 1 h at 37 °C in the dark. Cell nuclei were stained with 1 μg/ml DAPI for 5 min. Fluorescent images were captured using a Zeiss LSM980 Confocal Laser Microscope System. TUNEL-positive cells (green fluorescence) and total nuclei (blue) were counted using ImageJ. Apoptotic rate was expressed as the percentage of TUNEL-positive cells relative to total nuclei.

### Quantitative real-time PCR

Total RNA was extracted from the cells using TRIzol reagent (Invitrogen, 15596026). Reverse transcription of total RNA and cDNA amplification were performed as previously described^35^. The gene expression levels were calculated using the 2^−ΔΔCT^ method. The data were presented as fold changes relative to GAPDH. The primer sequences used in the study are listed in Table 1.

**Table 1:** List of porcine and human primers used in the study.

Genes	Primer sequences (5′–3′)
CD44 (porcine)	F: ACCCCAGCTACCAATGAGG
	R: GCCACTGGATGGTCTTTGTC
CXCL2 (porcine)	F: ATGTGTTTGGCAGGTCTCTGT
	R: CACAGTGGATGGTACAGCGT
CXCL8 (porcine)	F: AAGTGGGTGCAGAAGGTTG
	R: AAATTCTTGGGAGCCACGGA
MMP3 (porcine)	F: AGTAACGCTTTGATGTACCCAG
	R: AAGGACAAAGCAGGATCACA
MMP12 (porcine)	F: CAGCCATAATGTTCCCGTA
	R: CAGATGGTAAGTTTGCACCTA
PTGS2 (porcine)	F: TTAAACAGGAGCACCCGGAA
	R: CATCAATCTGGAAGGCGTCA
CD44 (human)	F: CCCCAGCAACCCTACTGATG
	R: CCCATGTGAGTGTCTGGTAGC
GAPDH	F: ACAACTTTGGTATCGTGGAAGG
	R: GCCATCACGCCACAGTTTC

### Western blotting

Cells were lysed using RIPA buffer (Beyotime, P0013B) for 30 min on ice. Cell extracts were obtained by centrifugation at 12,000*g* at 4 °C for 15 min, and the supernatant was collected and subjected to protein quantification as described previously^35^. Approximately 20 μg proteins was subjected to SDS–PAGE gel electrophoresis and transferred to nitrocellulose membranes (Bio-Rad). After blocking with 5% skim milk, the membranes were incubated with primary antibodies, including MMP3 (Biorbyt, orb11062), COL2 (Abcam, ab34712), CD44 (Abcam, ab157107), NF-κB p65 (CST, 6956S), p-NF-κB p65 (CST, 3033S), p38 MAPK (CST, 8690T), p-p38 MAPK (CST, 4511T), JNK (Proteintech, 66210-1-Ig), p-JNK (80024-1-RR), c-fos (CST, 2250T), p-c-fos (CST, 5348T), c-Jun (Biorbyt, orb338983), p-c-Jun (CST, 3270T), PTD-p65-P1 peptide (GlpBio, GC38520) and GAPDH (Abcam, ab8245) overnight at 4 °C. After incubation with secondary antibodies, protein bands were visualized with chemiluminescence (Tanon) and quantified using ImageJ. Protein expression levels were normalized to the internal control GAPDH, and relative expression was calculated by dividing the target protein intensity by the GAPDH intensity.

### Cell immunofluorescence

Cells were cultured on glass coverslips at 2 × 10^5^ per well in 24-well plates. Cells were fixed with 4% formaldehyde solution (Thermo Fisher) for 10 min, permeabilized with 0.2% Triton X-100 (BioFroxx, 1139ML100) for 5 min and blocked with 1% BSA (Sigma, A8806) for 1 h. The cells were incubated with phalloidin (Invitrogen, A34055) at room temperature for 1 h. Cells were then incubated with CD44 primary antibodies (Abcam, Ab157107) overnight at 4 °C and secondary antibodies (Invitrogen, A11055) for 1 h at room temperature. Next, the cells were incubated with DAPI-containing fluorescence mounting medium (SouthernBiotech, 010020) and examined by Zeiss LSM980 Confocal Laser Microscope System. Cell area, circularity and fluorescence intensity were quantified using ImageJ analysis software. Circularity was used to describe the roundness of the cell, with lower values indicating either an elongated shape or an increase in cell protrusions^36^. Cell circularity is mathematically defined as 4π*A*/*P*^2^, where *A* represents the area of the cell and *P* denotes its perimeter. Phalloidin-stained images were converted to 8-bit grayscale and thresholded to define cell boundaries. Individual cells were selected using the ‘Wand Tool’ to automatically trace cell contours. The ‘Analyze Particles’ tool measured *A* and *P*, with circularity values ranging from 0 (elongated) to 1 (perfect circle). For fluorescence intensity quantification, raw images were converted to 8-bit grayscale. Individual cell boundaries were delineated with the freehand selection tool, and mean fluorescence intensity was measured for individual cells.

### Atomic force microscopy

Atomic force microscopy (AFM; JPK NanoWizard II) analysis was performed as previously described^37^. In brief, the AFM was mounted on an inverted optical microscope (Nikon eclipse Ti-U, Nikon) and operated in contact mode with a loading rate of 0.4 μm/s and set point in 0.3 V. A silicon nitride cantilever tip (CSG01, NT-MDT) was driven to approach the cell surface and held for 30 s, and the end of the tip was cut off using focused ion-beam milling (FEI Quanta 200 3D FIB/SEM) to form a flat-ended cylindrical tip with a diameter of 3 μm before used^38^. The tip was then unloaded at the same loading rate, and the data were collected simultaneously during the entire indentation. Quantitative analysis of acquired data was performed using the JPK data processing software from the AFM manufacturer. To estimate the viscoelastic of the cells, the indentation curves were fitted using the rate-jump method^39^. The Young modulus was calculated using the following formula:

$$\Delta {\dot{\rm{P}}}=2{\rm{a}}\left(\frac{{\rm{E}}}{1-{{\rm{\nu }}}^{2}}\right)\Delta {\dot{{h}}},$$

where *h* and *P* are the indentation depth and indentation force, respectively; *E* and *ν* are the sample’s intrinsic Young’s modulus and Poisson’s ratio, respectively; and *a* is the radius of the cylindrical end of the tip. $\Delta \dot{{{P}}}$ and $\Delta \dot{{{h}}}$ are jumps in the rates of *P* and *h*, respectively, across the unloading point.

### RNA-seq and bioinformatics analysis

Total RNA was extracted using TRIzol reagent (Invitrogen) from porcine chondrocytes in different treatment groups. RNA samples were sent to Gene Denovo Biotechnology, for conducting RNA sequencing (RNA-seq) analysis on the Illumina HiSeq2500 platform. The reference genome used in this study was obtained from the Ensembl database (version: Ensembl 96, species: *Sus scrofa*, genome assembly: Sscrofa11.1). The data were accessed via the Ensembl website (https://www.ensembl.org). The mapped reads of each sample were assembled using StringTie v1.3.1 in a reference-based approach^40,41^. For each transcription region, a fragment per kilobase of transcript per million mapped reads (FPKM) value was calculated to quantify its expression abundance and variations, using RSEM software^42^. Transcripts with log_2_|fold change| >1 and false discovery rate (FDR) <0.05 were considered differentially expressed. Volcano plot generation, Venn diagram analysis, and Kyoto Encyclopedia of Genes and Genomes (KEGG) pathway analysis were performed using the Omicsmart online platform (https://www.omicsmart.com) based on the screened-out DEGs. The original RNA-seq data generated in this study have been deposited in the NCBI SRA database under BioProject accession number PRJNA1236871 (https://www.ncbi.nlm.nih.gov/bioproject/PRJNA1236871/). For the analysis of the published database on OA human knee cartilage tissue and healthy controls, KEGG pathway analysis was performed using the Omicsmart online platform, based on DEGs obtained from GSE113825 (obtained from supplementary table 4 in ref. ^43^) and GSE114007 (obtained from supplementary table 1 in ref. ^44^), respectively.

### Chondrocyte treatment with CD44 ligands

The following CD44 ligands, including A6 peptide (MCE, HY-P2230), low-molecular-weight hyaluronan (LMWHA, Sigma, 75046) and high-molecular-weight hyaluronan (HMWHA, Sigma, 53747), were used in this study. In most experiments, cells were treated with EPA for 8 h and then stimulated with 10 μM A6 peptide or 1 mg/ml LMWHA or 1 mg/ml HMWHA for 30 min. In some experiments, cells were treated with 10 μM A6 peptide and 1 mg/ml HMWHA or 1 mg/ml LMWHA and 1 mg/ml HMWHA for 30 min. In other experiments, cells were stimulated with 10 ng/ml IL-1α for 24 h and then treated with 1 mg/ml HMWHA for 30 min.

### Cartilage explant harvest and treatment with EPA and IL-1α

Porcine cartilage explants were obtained from the metacarpophalangeal joints of pigs. In brief, cylindrical cartilage explants (3 mm in diameter and 1 mm in thickness) were collected using a biopsy punch (Miltex). The explants were washed with phosphate-buffered saline (PBS) and incubated in the DMEM containing 1% penicillin–streptomycin solution for 48 h. The explants were then transferred to a 24-well plate and cultured in the medium supplemented with 30 µg/ml EPA and 10 ng/ml IL-1α. The method for preparation of human OA cartilage explants was similar to the preparing porcine cartilage explants. Human OA cartilage explants were treated only with 30 µg/ml EPA.

### Sulfated glycosaminoglycan (sGAG) assay

Porcine cartilage explants were cultured in medium supplemented with 30 µg/ml EPA and 10 ng/ml IL-1α for 10 days. The culture medium was changed and collected every other day. Dimethylmethylene blue (Sigma, 341088) assay^45^ was used to determine the sGAG release in the culture medium due to breakdown of cartilage ECM. Different concentrations of chondroitin sulfate (Sigma, C9819) were used to plot a standard curve.

### Nanoindentation test

The Young’s modulus of porcine cartilage explants was measured using the nanoindentation (Piuma Chiaro, Optics11). A spherical nanoindentation probe with a radius of 9 μm and a stiffness of 262.7 N/m was used. Explants were attached to 6-cm dishes containing PBS at room temperature and indented at a loading rate of 2 μm/s. The tip was held in this indentation depth for 10 s. After the holding, the tip was unloaded at the same loading rate. The indentation curves were fitted using the rate-jump method. The Young’s modulus of explants was calculated as previously described in the ‘Atomic force microscopy’ section.

### Synthesis of EPA emulsion and EPA emulsion-integrated HA injection

EPA emulsion was prepared through ultrasonic emulsification. EPA (Sigma, ≥99%, 10417-94-4) and 1, 2-Distearoyl-sn-glycero-3-phosphoethanolamine-Poly (ethylene glycol) (DSPE-PEG) (molecular weight 2,000 Da, Aladdin, 147867-65-0) were co-dissolved in dichloromethane (GR, Macklin) at a fixed mass ratio of 5:1. Then, 100 μl of EPA/DSPE-PEG solution was added dropwise to 5 ml aqueous dispersion phase under agitation. Next, the mixed suspension was emulsified by tip sonication (ultrasonic cell disruptor, SCIENTZ, power: 100%; mode: 2 min working time with 2 s ON and 5 s OFF intervals) in an ice bath. Subsequently, the organic solvent was completely removed using a rotary evaporator (IKA) at 40 °C under reduced pressure, stepped down from 550 mbar to 10 mbar, yielding the EPA emulsion. The concentration of EPA emulations can be adjusted to 2–50 mg/ml with desired size distribution and colloidal stability. The EPA emulsion was filtered through a 0.22-μm filter membrane and stored under aseptic conditions at 4 °C. Before use, the EPA emulsion (50 mg/ml) and clinical-grade hyaluronic acid (HA) injections (molecular weight 600–1,170 kDa; ARTZ Dispo, Seikagaku Corporation) were mixed at a volume ratio of 1:9 to obtain EPA emulsion-integrated HA injections (EPA 5 mg/ml).

### Dynamic light scattering

The size and size distribution of EPA emulsions were characterized by dynamic light scattering analysis (Nicomp 380 N3000, PSS). Measurements were performed after dilution, using 488-nm incident light and at a measuring angle of 90° at 25 °C.

### Rheological analysis

The rheological properties of EPA emulsion-integrated HA injections were studied using a stress-controlled rheometer (Kinexus, Malvern). The dynamic viscosity of HA injections with and without EPA emulsion was measured with 20-mm cone and plate (1° cone angle) at shear rates from 0.01 to 1,000 s^−1^. Similarly, dynamic viscoelasticity was measured over an angular frequency range of 0.1 to 100 Hz at a fixed shear strain of 1%.

### Experimental OA model and intraarticular injection in mice

All animal experiments were approved by the Institutional Animal Care and Use Committee of Guangzhou Medical University (no. S2020-140). This study was reported in accordance with the ARRIVE guidelines 2.0. Sixty Male C57BL/6J mice (aged 10–12 weeks, weighing 30 ± 2 g) were obtained from Guangdong Medical Laboratory Animal Center (Guangzhou, China). The mice were housed with an average of six per cage and had free access to food and water. Anterior cruciate ligament transection (ACLT) surgeries were performed to induce posttraumatic OA as previously described^46^. In brief, mice were anesthetized by inhalation of isoflurane, and ACLTs were performed by transecting the anterior cruciate ligament in the right knee joints using a surgical microscope. Sham surgeries were performed using the same approach, but without ACLT. The mice were randomly divided into five groups (12 mice per group): sham, ACLT injected with PBS (ACLT), ACLT injected with HA solution (HA), ACLT injected with EPA emulsion (EPA) and ACLT injected with EPA emulsion-integrated HA injection (EPA–HA). Random numbers were generated in Excel using the RAND function. Intraarticular injections were initiated 14 days after ACLT surgery. A total of four weekly injections (10 μl per dose) of PBS, EPA emulsion, HA or EPA emulsion-integrated HA solution were administered into the knee joint cavity at 2, 3, 4 or 5 weeks after surgery. Four or 8 weeks after surgeries, six mice in each group (*n* = 6) were euthanized, and their knee joints were collected for histological and immunohistochemical analysis. None of the mice exhibited wound infection, knee joint infection or mortality. Sample size was calculated using the resource equation approach^47^, which indicated that an *n* of six is sufficient for our histological studies.

### Micro-CT analysis

Mouse knee joints were scanned using the Skyscan 1172 micro-computed tomography (micro-CT) scanner (Skyscan 1172, Bruker). Sagittal views of the knee joint were reconstructed using NRecon software (v2.0.4.0, Skyscan) and then visualized using Dataviewer software (v1.5.6.2, Bruker). The subchondral bone of the medial tibial plateau was identified as the region of interest and quantified using CT Analyzer software (v1.13, Skyscan) for trabecular bone volume per tissue volume (BV/TV) and trabecular bone pattern factor (Tb.Pf).

### Histological and immunohistochemical staining

Samples from mouse, porcine and human OA cartilage were fixed in 4% paraformaldehyde for 24 h, decalcified in 0.5 M ethylenediaminetetraacetic acid (EDTA) for 3 weeks, embedded in paraffin and sectioned at a thickness of 6 μm. Tissue slides were stained with hematoxylin and eosin (H&E) to evaluate cartilage thickness or Safranin O/Fast Green to assess cartilage degradation using the Osteoarthritis Research Society International (OARSI) score system^48^. The boundaries of the hyaline cartilage (HC) and calcified cartilage (CC) regions were identified using the tidemark and subchondral bone lines in HE cartilage sections. HC was defined as the nonmineralized cartilage layer above the tidemark, while CC comprised the mineralized tissue between the tidemark and subchondral bone line. Cartilage thickness is defined as the sum of HC and CC. Sagittal sections were divided into anterior, middle and posterior regions. Three measurements per region (nine points per section) were averaged across six nonconsecutive sections per joint to minimize regional bias. For immunohistochemistry, the sections were blocked with 3% BSA for 30 min at room temperature and incubated with CD44 primary antibodies overnight at 4 °C. After washing, the sections were incubated with secondary antibodies and visualized using 3,3′-diaminobenzidine-peroxidase substrate solution.

### Statistical analysis

All statistical analyses were performed using GraphPad Prism 8 (GraphPad Software). Data are presented as mean ± standard deviation (s.d.). Statistical analysis was performed using unpaired Student’s *t*-test or one-way analysis of variance (ANOVA) followed by Tukey’s multiple-comparisons test. All tests were two-tailed. Differences were considered statistically significant at *P* < 0.05.

## Results

### EPA regulates F-actin organization and cell mechanical properties in IL-1α-treated porcine chondrocytes and human OA chondrocytes

**Fig. 1:**
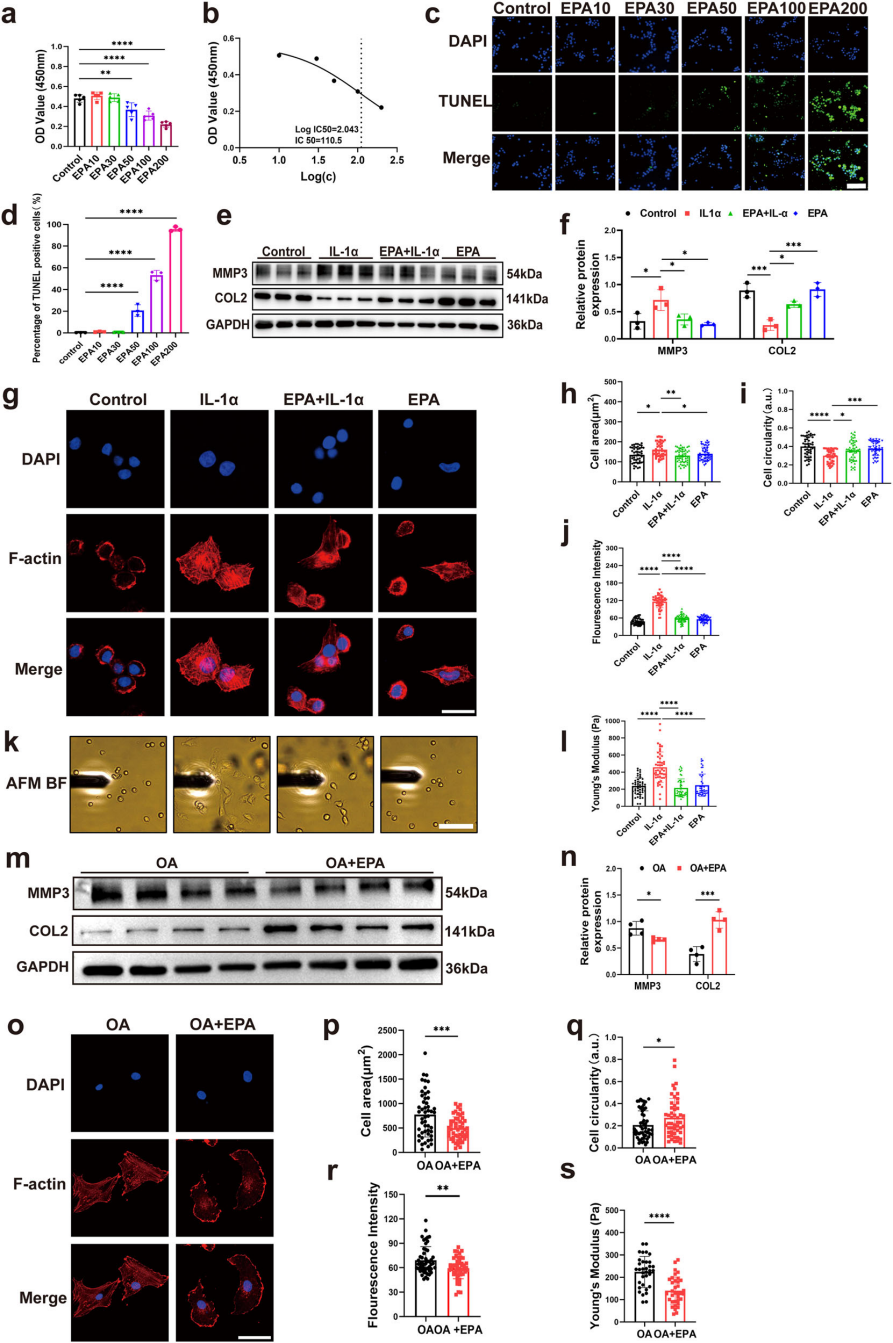
EPA regulates F-actin organization and cell mechanical properties in IL-1α-treated porcine chondrocytes and human OA chondrocytes. **a** Primary porcine chondrocytes were treated with varying concentrations of EPA for 48 h and subjected to CCK-8 analysis. **b** IC_50_ of EPA on chondrocytes. Concentrations were converted to log(*c*). The vertical dotted line indicates the IC_50_, with an associated OD value of 2.046. Primary porcine chondrocytes were pretreated with or without EPA for 8 h and then stimulated with IL-1α for 24 h. **c** Representative TUNEL staining images of chondrocytes treated with EPA (0–200 μg/ml) for 48 h. **d** Quantitative analysis of TUNEL-positive cells. Data are presented as the percentage of TUNEL-positive chondrocytes as a propotion of total cells. **e**, **f** Representative images of western blot gel (**e**) and quantification (**f**) of MMP3 and COL2 in porcine chondrocytes. **g** Representative immunofluorescence images of F-actin and quantification of cell area (**h**), cell circularity (**i**) and fluorescence intensity (**j**) of F-actin. **k** Representative bright-field images showing that porcine chondrocytes were tested with an AFM probe. **l** Quantification of Young’s modulus. Primary human OA chondrocytes were treated with or without EPA for 8 h. **m**, **n** Representative images of western blot gel and quantification of MMP3 and COL2 in human OA chondrocytes. **o** Representative immunofluorescence images of F-actin and quantification of cell area (**p**), cell circularity (**q**), fluorescence intensity of F-actin (**r**) and Young’s modulus (**s**). Scale bars, 50 μm (**c**), 20 μm (**g**), 100 μm (**k**) and 20 μm (**o**). Data are presented as mean ± s.d. *n* = 5 (**a**), 3 (**d**), 3 (**f**), 4 (**n**) and 50 (**h**–**j**, **l** and **p**–**s**). Statistical analysis was performed using one-way ANOVA with Tukey’s multiple-comparisons test in **a**, **d**, **f** and **h**–**l,** or unpaired Student’s *t*-test in **n** and **p**–**s**. *P < 0.05, **P < 0.01, ***P < 0.001.

To determine the optimal dose for the in vitro studies, the cytotoxicity of EPA on porcine chondrocytes was assessed using a CCK-8 assay. Chondrocytes were treated with varying concentrations of EPA (0, 10, 30, 50, 100 and 200 μg/ml) for 48 h. Compared with the control group, 50, 100 and 200 μg/ml of EPA reduced the optical density (OD) value of cells (Fig. 1a). The half-maximal inhibitory concentration (IC_50_) of EPA was 110.5 μg/ml (Fig. 1b). These results indicated that incubating chondrocytes in 10 and 30 μg/ml EPA for 48 h did not cause apparent toxicity compared with the control group. In addition, the percentage of TUNEL-positive cells showed a significant increase starting at 50 μg/ml EPA, with further elevation at 100 and 200 μg/ml (Fig. 1c, d). These results align with the CCK-8 viability data and confirm that the cytotoxicity of EPA at ≥50 μg/ml is at least partially mediated by apoptosis induction, particularly in the late apoptotic phase. Previous studies have reported that 30 μg/ml EPA was more effective in reducing matrix metalloproteinases (MMPs) than 10 μg/ml EPA^5^. Therefore, a final concentration of 30 μg/ml EPA was selected for the following in vitro studies. Next, we verified the effectiveness of IL-1α treatment in porcine chondrocytes. Western blot results showed that IL-1α increased MMP3 expression and decreased type II collagen (COL2) expression at the protein level (Fig. 1e, f). These effects were reduced by combined treatment with EPA and IL-1α (Fig. 1e, f).

To investigate the effects of EPA on chondrocyte mechanics, we began by using phalloidin staining to observe alterations in chondrocyte morphology and F-actin organization. IL-1α-treated chondrocytes exhibited a more spindle-like shape compared with control chondrocytes (Fig. 1g) as indicated by increased cell area and decreased cell circularity (Fig. 1h, i). In addition, IL-1α-treated chondrocytes showed more pronounced F-actin stress fibers and greater F-actin intensity compared with control chondrocytes (Fig. 1g). Combined treatment with EPA and IL-1α restored chondrocytes to a rounded morphology with a cortical F-actin organization (Fig. 1g) and decreased F-actin intensity (Fig. 1j). AFM analysis showed that IL-1α increased the Young’s modulus of porcine chondrocytes, whereas this effect was blocked by combined treatment with EPA and IL-1α (Fig. 1k, l). Next, we examined the effects of EPA on freshly isolated human OA chondrocytes. Western blot results showed that EPA downregulated MMP3 and upregulated COL2 protein levels in human OA chondrocytes (Fig. 1m, n). In addition, EPA-treated human OA chondrocytes showed increased circularity, decreased cell area, F-actin intensity and Young’s modulus compared with control chondrocytes (Fig. 1o–s).

### EPA reduces CD44 expression in IL-1α-treated porcine chondrocytes and in human OA chondrocytes

**Fig. 2:**
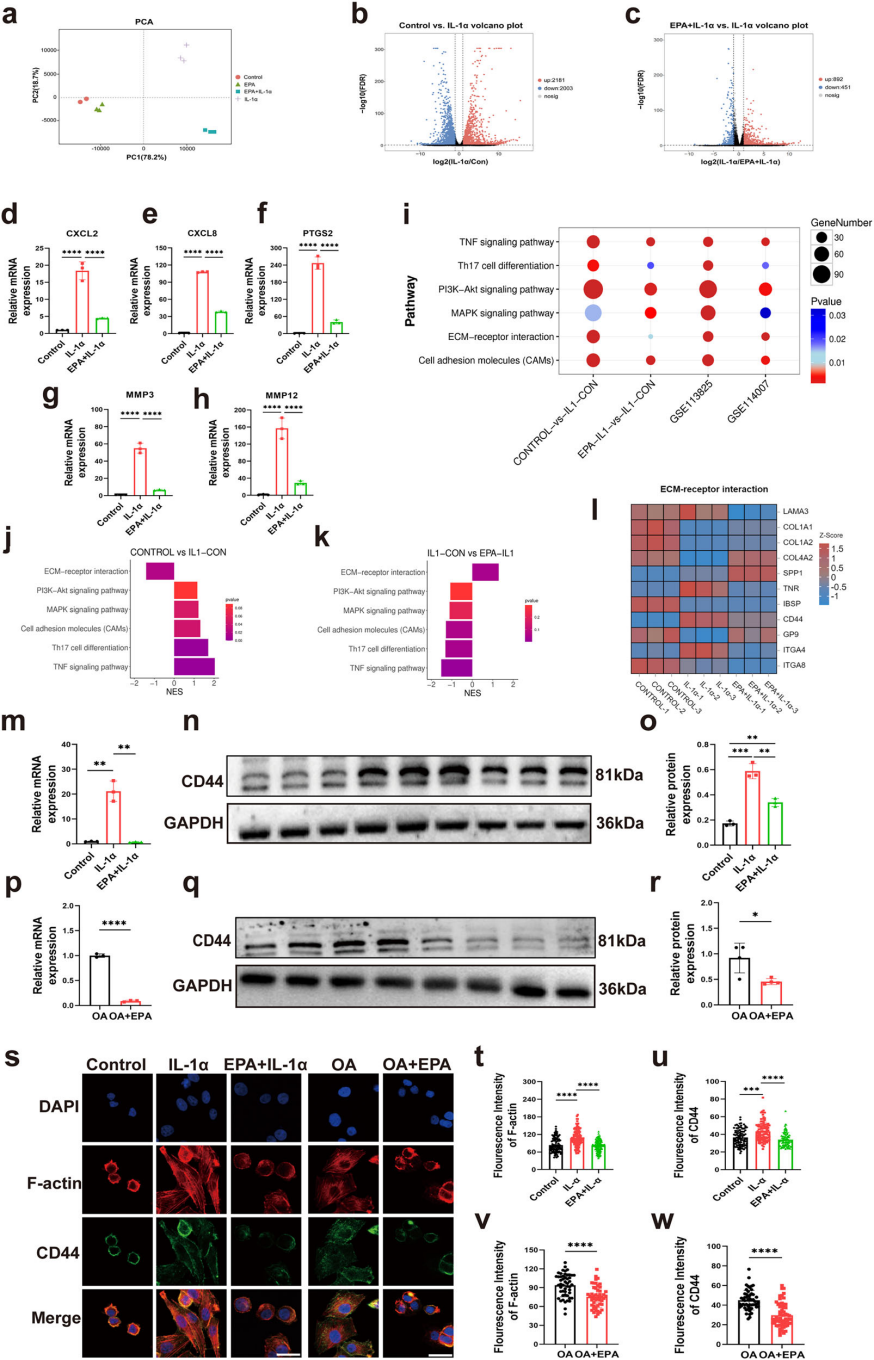
EPA reduces CD44 expression in IL-1α-treated porcine chondrocytes and in human OA chondrocytes. **a**–**c** PCA plot of RNA-seq samples (**a**) and volcano maps of DEGs generated from porcine chondrocytes (**b** and **c**). Values of FDR <0.05 and log_2_|fold change| >1 were accepted as significant. **d**–**h** Gene expression levels of CXCL2 (**d**), CXCL8 (**e**), MMP3 (**f**), MMP12 (**g**) and PTGS2 (**h**) in porcine chondrocytes. **i** KEGG bubble plot showing enriched pathways across experimental and human OA datasets. **j**, **k** GSEA plots illustrating directional regulation of representative pathways. **l** Heatmap of DEGs in the ECM–receptor interaction pathway in porcine chondrocytes. **m**–**r** Gene expression level, representative images of western blot gel and protein expression level of CD44 in porcine chondrocytes and human OA chondrocytes. **s**–**w** Representative co-immunostaining images (**s**) and fluorescence intensity (**t**–**w**) of F-actin and CD44 in porcine chondrocytes and human OA chondrocytes. Scale bar, 20 μm. Data are presented as mean ± s.d. *n* = 3 (**d**–**h**, **m**, **o** and **p**), 4 (**r**), 100 (**t** and **u**) and 50 (**v** and **w**). Statistical analysis was performed using one-way ANOVA with Tukey’s multiple-comparisons test in **d**–**h**, **m**, **o**, **t** and **u**, or unpaired Student’s *t*-test in **p**, **r**, **v** and **w**. *P < 0.05, **P < 0.01, ***P < 0.001.

To explore how EPA regulates chondrocytes mechanics, we performed RNA-seq in porcine chondrocytes. The gene expression principal component analysis (PCA) plot showed that samples in control, IL-1α and EPA + IL-1α groups were located distinct from each other, while samples in control group were located close to samples in EPA (Fig. 2a). Under the criteria of FDR <0.05 and log_2_|fold change| >1, 4,184 DEGs were found between control and IL-1α groups (Fig. 2b), while 1,343 DEGs were found between IL-1α and EPA + IL-1α groups (Fig. 2c). Next, quantitative real-time PCR was used to validate the RNA-seq results. Gene expression levels of CXCL2, CXCL8, MMP3, MMP12 and PTGS2 increased after IL-1α stimulation, and this increase was inhibited by combined treatment with EPA and IL-1α (Fig. 2d–h). We then performed separate KEGG analyses for each dataset: control versus IL-1α, and IL-1α versus EPA-IL-1α, GSE113825 and GSE114007. The results identified six common pathways across all datasets, including TNF signaling pathway, Th17 cell differentiation, PI3K–Akt signaling pathway, MAPK signaling pathway, ECM–receptor interaction and cell adhesion molecules (Fig. 2i). Gene set enrichment analysis (GSEA) further revealed directional regulation: IL-1α activated TNF, MAPK, PI3K-Akt, Th17 and cell adhesion pathways but suppressed ECM–receptor interaction (Fig. 2j). EPA treatment mitigated these effects, normalizing pathway activity toward baseline (Fig. 2k). Notably, ECM–receptor interaction pathway was ranked 1st, 26th, 15th and 2nd among enriched pathways in these four sets of DEGs, respectively. Among these candidate genes in the ECM–receptor interaction pathway, including LAMA3_5, COL1A, COL4A, SPP1, TN, IBSP, CD44, GP9, ITGA4 and ITGA8 (Fig. 2l), we found that CD44 gene, a major surface HA receptor and a key F-actin organizer, was also upregulated in OA samples in GSE113825. IL-1α increased CD44 gene expression and protein levels in porcine chondrocytes, and this effect was inhibited by combined treatment with EPA and IL-1α (Fig. 2m–o). In human OA chondrocytes, CD44 gene expression and protein levels was also decreased by EPA (Fig. 2p–r). Co-immunostaining F-actin and CD44 showed that EPA restored IL-1α-treated porcine chondrocytes and human OA chondrocytes to a rounded morphology with a cortical F-actin and CD44 organization (Fig. 2s), and decreased F-actin and CD44 intensity (Fig. 2t–w).

### EPA reduces CD44 expression by inhibiting the NF-κB p65 signaling pathway in IL-1α-treated porcine chondrocytes

**Fig. 3:**
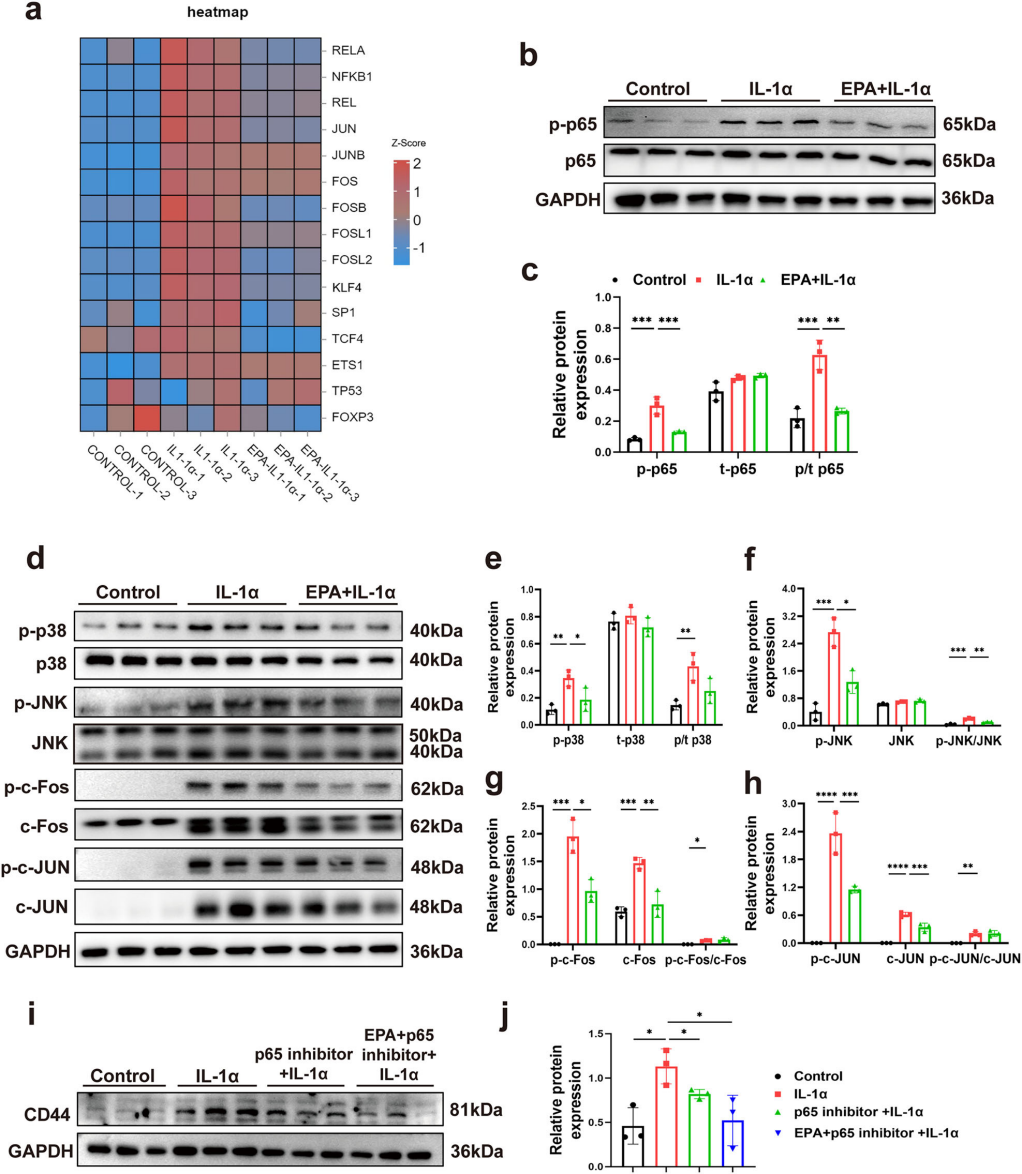
EPA reduces CD44 expression by inhibiting the NF-κB p65 signaling pathway in IL-1α-treated porcine chondrocytes. **a** Heatmap showing the expression of TFs in porcine chondrocytes under different treatment conditions. **b**, **c** Representative images of western blot gel (**b**) and protein expression levels (**c**) of p-p65, t-p65 and p-p65/t-p65 in porcine chondrocytes. **d**–**h** Representative images of western blot gel (**d**) and protein expression levels of p-p38, t-p38, p-p38/t-p38 (**e**), p-JNK, JNK, p-JNK/JNK (**f**), p-c-Fos, c-Fos, p-c-Fos/c-Fos (**g**), p-c-Jun, c-Jun and p-c-Jun/c-Jun (**h**) in porcine chondrocytes. **i**, **j** Representative images of western blot gel (**i**) and protein level (**j**) of CD44 in porcine chondrocytes treated with p65 inhibitor. Data are presented as mean ± s.d. *n* = 3. Statistical analysis was performed using one-way ANOVA with Tukey’s multiple-comparisons test in **c** and **e**–**j**. *P < 0.05, **P < 0.01, ***P < 0.001.

Next, we aimed to investigate the mechanism by which EPA inhibited CD44 expression in IL-1α-treated porcine chondrocytes. Previous studies have shown that CD44 promoter activity is regulated by various TFs, including both positive regulators (for example, Sp1, TCF4, AP-1, NF-κB and ETS-1) and negative regulators (for example, p53, KLF4 and Foxp3) (reviewed in ref. ^49^). KEGG analysis showed that, among these candidate TFs, the gene expression level of NF-κB (RELA, NFKB1 and REL), AP-1 (JUN, JUNB, FOS, FOSB, FOSL1 and FOSL2) and KLF4 was significantly increased after IL-1α treatment. This increase was significantly inhibited by the combined treatment of EPA and IL-1α (Fig. 3a). We then verified the effect of EPA on IL-1α-induced activation of NF-κB and AP-1. Western blot results showed that IL-1α did not affect total p65 expression, but it significantly increased p-p65 expression and elevated the ratio of p-p65 to p65 in comparison with the control (Fig. 3b, c). Combined treatment of EPA and IL-1α blocked the upregulation of p-p65 and reduced the ratio of p-p65 to p65 in comparison with the IL-1α stimulation (Fig. 3b, c). In addition, IL-1α significantly increased expression of p-p38 and p-JNK and elevated the ratio of p-p38 to p38 and the ratio of p-JNK to JNK in comparison with the control, while combined treatment of EPA and IL-1α significantly inhibited these effects (Fig. 3d–f). IL-1α significantly increased the expression of p-c-Fos, c-Fos, p-c-JUN and c-JUN, and elevated the ratio of p-c-Fos to c-Fos and the ratio of p-c-JUN to c-JUN compared with the control (Fig. 3d, g, h). However, combined treatment with EPA and IL-1α did not reduce the ratio of p-c-Fos to c-Fos or p-c-JUN to c-JUN compared with IL-1α stimulation (Fig. 3g, h). These results suggest that EPA inhibited the activation of NF-κB P65 but not the MAPK–AP-1 signaling pathway in IL-1α-treated porcine chondrocytes. Furthermore, the p65 inhibitor PTD-p65-P1 was used to confirm that EPA decreased CD44 expression by inhibiting the phosphorylation of p65. Western blots results showed that p65 inhibitor or combined treatment of p65 inhibitor and EPA significantly decreased CD44 expression in IL-1α-treated porcine chondrocytes (Fig. 3i, j). There was no significant difference in CD44 expression between p65 inhibitor and combined treatment of p65 inhibitor and EPA groups (Fig. 3i, j). These results suggest that EPA decreased CD44 expression by inhibiting the phosphorylation of p65 in IL-1α-treated porcine chondrocytes.

### EPA ameliorates cartilage degeneration and decreases the expression of CD44 and p-p65 in IL-1α-treated porcine cartilage explants and human OA cartilage explants

**Fig. 4:**
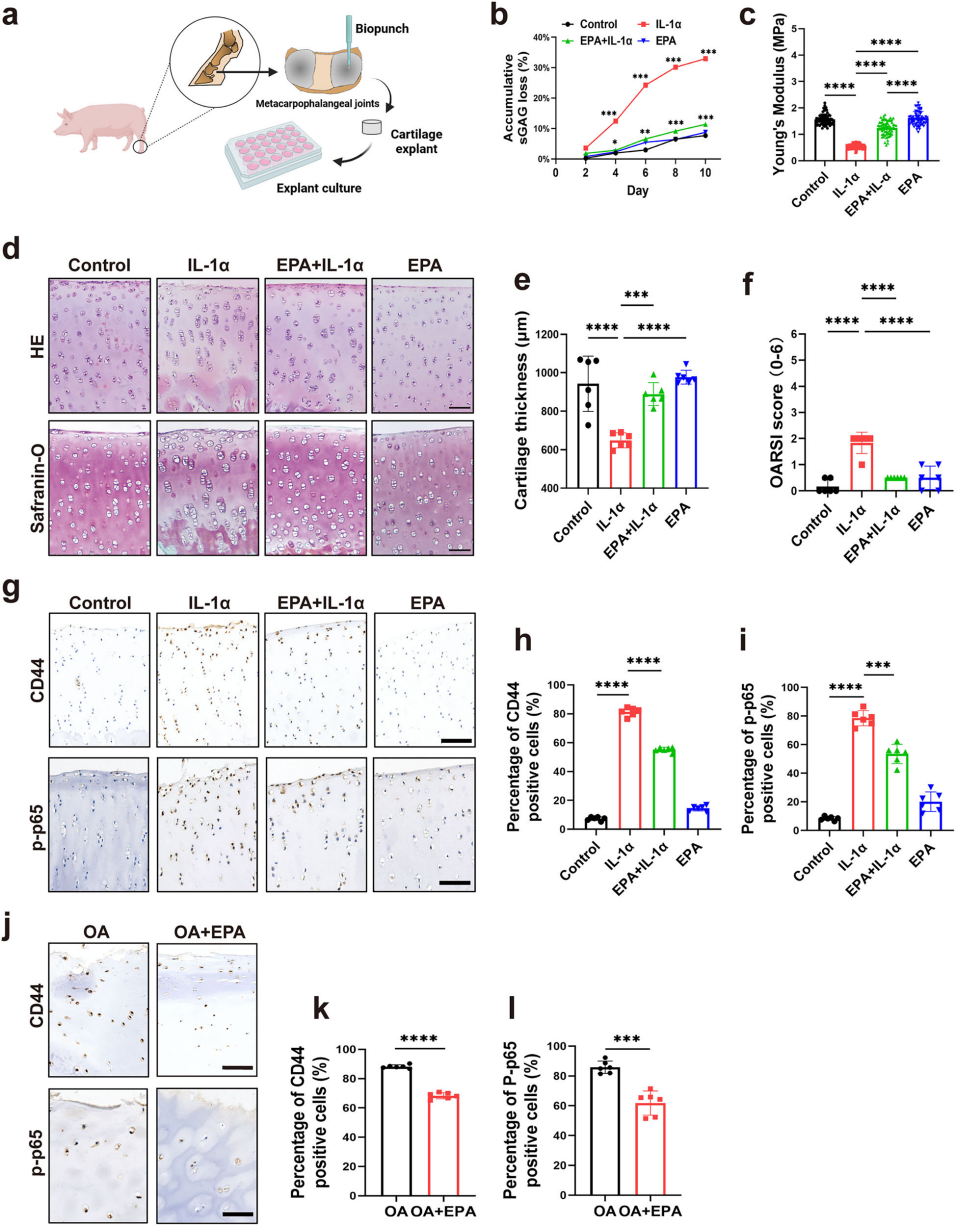
EPA ameliorates cartilage degeneration and decreases the expression of CD44 and p-p65 in IL-1α-treated porcine osteochondral explants and in human OA cartilage explants. **a** A schematic illustration summarizing the experimental design for porcine osteochondral explants. Osteochondral explants were collected from porcine metacarpophalangeal joints using a biopsy punch. **b** Accumulated loss of sGAG content from porcine osteochondral explants during 10 days of IL-1α treatment. **c** Mechanical properties of porcine osteochondral explants after 8-day IL-1α treatment. **d**–**f**, Representative images (**d**) and quantification (**e** and **f**) of H&E staining (top) and Safranin O/Fast Green staining (bottom) in porcine osteochondral explants under different treatment conditions. **g**–**i** Representative images of immunohistochemical staining (**g**) of CD44 and p-p65 in porcine osteochondral explants under different treatment conditions, and quantification (**h** and **i**) of CD44- and p-p65-positive chondrocytes as a proportion of total chondrocytes. **j**–**l**, Representative images of immunohistochemical staining (**j**) of CD44 and p-p65 in human OA cartilage explants treated with or without EPA, and quantification (**k** and **l**) of CD44- and p-p65-positive chondrocytes as a proportion of total chondrocytes. Scale bars, 200 μm (**d**, **g** and **j**). Data are presented as mean ± s.d. *n* = 3 (**b**), 75 (**c**) and 6 (**e**, **f**, **h**, **i**, **k** and **l**). Statistical analysis was performed using one-way ANOVA with Tukey’s multiple-comparisons test in **b**, **c**, **e**, **f**, **h** and **i**, or unpaired Student’s *t*-test in **k** and **l**. *P < 0.05, **P < 0.01, ***P < 0.001.

An ex vivo porcine cartilage explant model was used to corroborate the aforementioned findings (Fig. 4a). IL-1α treatment induced a stable sGAG loss of up to 33% by day 10, whereas combined treatment of EPA and IL-1α significantly reduced sGAG loss down to 11% (Fig. 4b). Young’s modulus and cartilage thickness were significantly decreased in the IL-1α group after 10 days, and this effect was significantly mitigated in the EPA and IL-1α combined treatment group (Fig. 4c–e). OARSI scores showed that EPA ameliorated IL-1α-induced cartilage degeneration (Fig. 4d, f). In addition, the number of CD44- and p-p65-positive articular chondrocytes was markedly increased in IL-1α group compared with the control group, and this effect was significantly inhibited by the combined treatment with EPA and IL-1α (Fig. 4g–i). Next, we performed CD44 and p-p65 staining in human OA cartilage explants from patients with OA. The number of CD44- and p-p65-positive articular chondrocytes was significantly decreased after EPA treatment (Fig. 4j–l).

### EPA regulates F-actin organization and cell mechanical properties in CD44 ligand-treated porcine chondrocytes

**Fig. 5:**
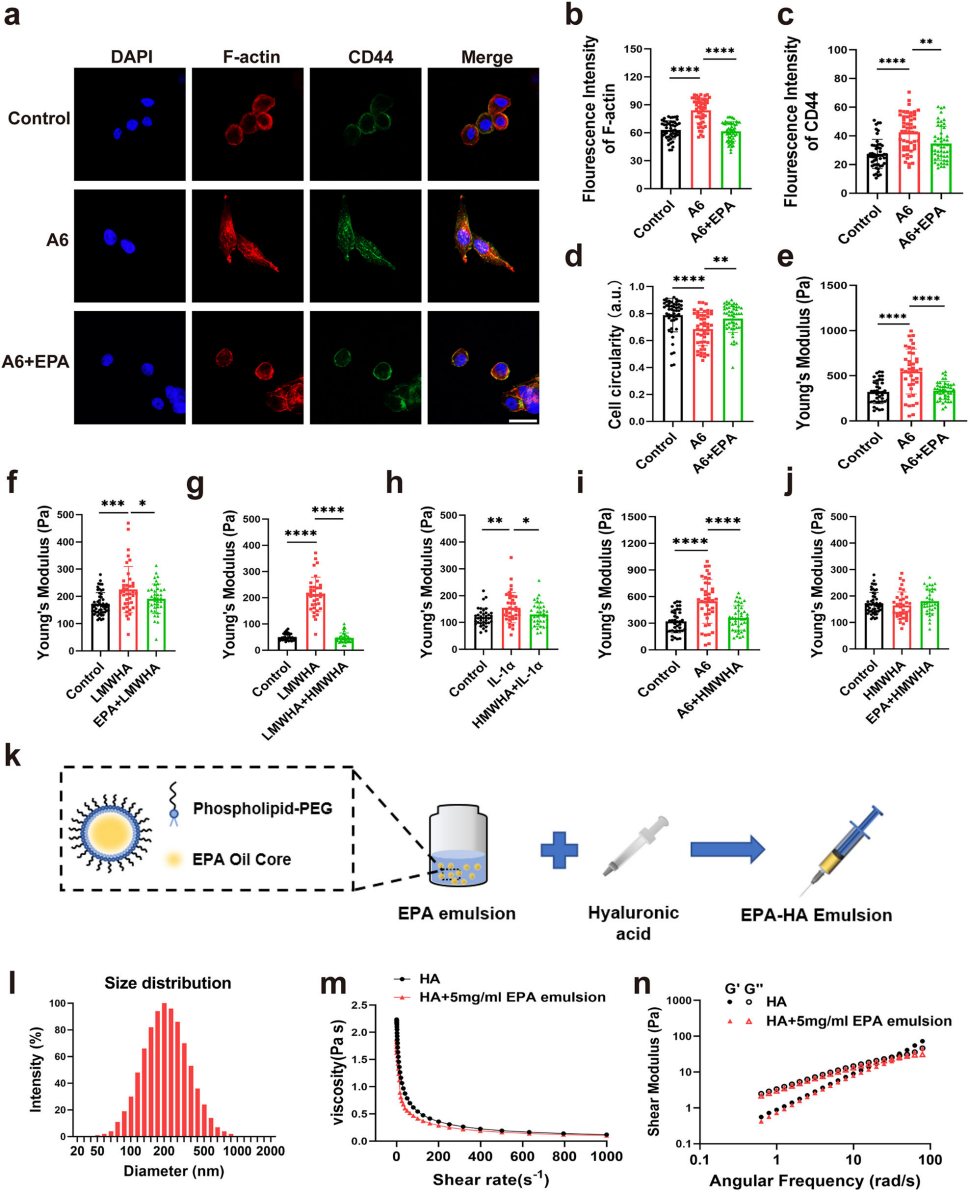
EPA regulates F-actin organization and cell mechanical properties in CD44 ligand-treated porcine chondrocytes. **a** Representative co-immunostaining images of F-actin and CD44 in A6-stimulated porcine chondrocytes pretreated with or without EPA. Scale bar, 20 μm. **b**–**d** Quantification of fluorescence intensity (**b** and **c**) of F-actin and CD44 and cell circularity (**d**) in A6-stimulated porcine chondrocytes pretreated with or without EPA. **e**–**j** Quantification of Young’s modulus in CD44 ligand-stimulated porcine chondrocytes pretreated with or without EPA. **k** A schematic illustration of EPA–HA injections. **l** Size distribution of EPA emulsion in aqueous suspensions. **m**, **n** Dynamic viscosity (**m**) and dynamic shear modulus (**n**) of intraarticular HA injections and EPA–HA injections. ●, storage modulus (G′) of HA; ○, loss modulus (G″) of HA; ▲, storage modulus (G′) of EPA–HA; △, loss modulus (G″) of EPA–HA. Data are presented as mean ± s.d. *n* = 50 (**b**–**d**) and 40 (**e**–**j**). Statistical analysis was performed using one-way ANOVA with Tukey’s multiple-comparisons test. *P < 0.05, **P < 0.01, ***P < 0.001.

Because elevated levels of CD44 do not necessarily correlate with increased CD44 activation and associated mechanical alterations, various CD44 ligands were used to investigate the effect of EPA on CD44-activated changes in chondrocyte mechanics. A6, an eight-amino-acid derived from human urokinase plasminogen activator, directly binds to and activates CD44. A6-stimulated chondrocytes exhibited more pronounced F-actin stress fibers, higher F-actin intensity and decreased cell circularity compared with control chondrocytes (Fig. 5a–d). Combined treatment with EPA and A6 restored chondrocytes to a rounded morphology with a cortical F-actin organization and reduced F-actin intensity (Fig. 5a–d). In addition, A6 increased the Young’s modulus of porcine chondrocytes (Fig. 5e). This effect was inhibited by combined treatment with A6 and EPA (Fig. 5e). HA, a major component of synovial fluid and cartilage, is the natural ligand of CD44. AFM analysis indicated that LMWHA (molecular weight <500 kDa) increased the Young’s modulus of porcine chondrocytes (Fig. 5f). This effect was significantly inhibited by combined treatment with EPA and LMWHA or combined treatment of LMWHA and HMWHA (molecular weight >1,000 kDa) (Fig. 5f, g). In addition, HMWHA also inhibited IL-1α- or A6-induced increases in chondrocyte stiffness (Fig. 5h, i). HMWHA or combined treatment with EPA and HMWHA had no effect on the Young’s modulus of porcine chondrocytes (Fig. 5j).

Next, EPA emulsion-integrated HA (EPA–HA) injections were developed for intraarticular application. EPA was first converted into highly stable nanoemulsions using DSPE-PEG, a Food and Drug Administration-approved emulsifier, and then blended with HA injections (Fig. 5k). Ultrasonic emulsification effectively produced uniformly sized nanoemulsions in the presence of DSPE-PEG. Dynamic light scattering results showed that, under optimized emulsification conditions, nanoemulsions with a hydrated particle size of approximately 200 nm and a high concentration of up to 50 mg/ml were obtained (Fig. 5l). To prepare EPA emulsion-integrated HA injections for further cell and animal experiments, the high-concentration EPA nanoemulsions were mixed with clinically used HA injections at a fixed volume ratio. To align with existing HA-based therapies while ensuring experimental reproducibility, we selected Artz, a clinically approved intraarticular injection with a molecular weight range of 600–1,170 kDa, to synthetic EPA–HA. Because synovial fluid naturally exhibits viscosity and viscoelasticity, clinically used HA injections are designed to mimic these physicochemical properties to maintain a normal joint environment. Therefore, we focused on characterizing the effects of the addition of the EPA emulsion on the physicochemical properties of the HA joint injection. Dynamic viscosity and rheological tests revealed that EPA emulsion-integrated HA injections prepared by mixing high-concentration EPA nanoemulsions at a lower volume ratio showed no significant differences in viscoelasticity and dynamic viscosity compared with clinical HA injections (Fig. 5m, n). This finding indicates that our formulation strategy achieves a high effective concentration of EPA (5 mg/ml) while maintaining physicochemical properties consistent with those of clinical HA intraarticular injections.

### EPA emulsion-integrated HA injection ameliorates ACLT-induced OA progression and decreases CD44 expression in mice

**Fig. 6:**
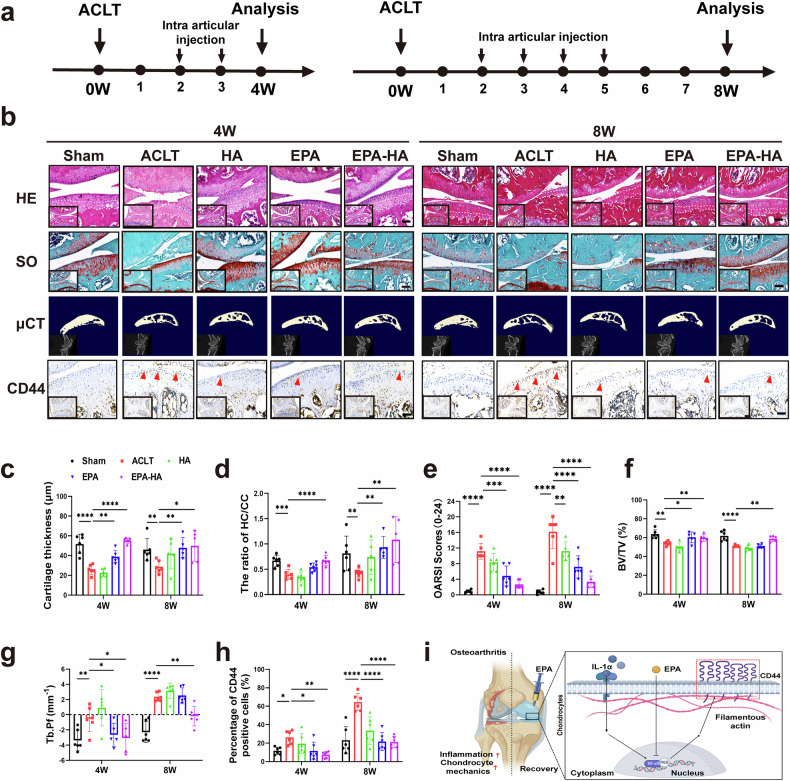
EPA emulsion-integrated HA injection ameliorates ACLT-induced OA progression and decreases CD44 expression in mice. **a** A schematic illustration showing the timeline for experiments in ACLT-induced OA mice. Animals were euthanized at either the 4th or 8th week after surgery, and knee joints were subsequently collected for histological and immunohistochemical analyses, respectively. **b**–**h** Representative images (**b**) and quantification (**c**–**h**) of H&E staining for each treatment group. The tidemark and subchondral bone line are demarcated with dotted white lines in the magnified histological images: representative images and quantification of Safranin O/Fast Green staining for each treatment group (**b** and **e**); representative images of micro-CT scans and quantification of BV/TV and Tb.Pf for each treatment group (**b**, **f** and **g**); and representative images and quantification of immunohistochemical staining of CD44 for each treatment group (**b** and **h**). Scale bars (**b**), 200 μm in original images amd 50 μm in magnified images. **i**, A schematic illustration summarizing the mechanism through which EPA inhibits the inflammation-induced increase in chondrocyte mechanics by suppressing the NF-κB p65/CD44 signaling pathway and alleviates OA. Data are presented as mean ± s.d. *n* = 6. Statistical analysis was performed using one-way ANOVA with Tukey’s multiple-comparisons test. *P < 0.05, **P < 0.01, ***P < 0.001.

The next series of experiments used a mouse ACLT-induced OA model to investigate the role of EPA emulsion or EPA emulsion-integrated HA injection in OA pathogenesis in vivo (Fig. 6a). H&E and Safranin O staining showed that the articular cartilage in the sham group had a smooth surface, normal thickness and intact structures at 4 and 8 weeks (Fig. 6b). H&E staining indicated that, compared with the sham group, the thickness of HC was significantly lower and the ratio of HC to CC was significantly reduced in the ACLT group at 4 and 8 weeks (Fig. 6c, d). This effect was markedly diminished in the EPA or EPA–HA group, but not in the HA group (Fig. 6c, d). Similarly, OARSI scores from Safranin O staining indicated that intraarticular injection of EPA or EPA–HA alleviated ACLT-induced articular cartilage degeneration at 4 and 8 weeks (Fig. 6e).

Micro-CT results revealed that, compared with the sham group at 4 weeks, BV/TV (reflecting trabecular bone mass) was significantly decreased in the ACLT and HA groups, while Tb.Pf (indicating disrupted trabecular connectivity and transition from plate-like to rod-like structure) values were significantly increased (Fig. 6f, g). This effect was significantly diminished in the EPA and EPA–HA groups (Fig. 6f, g). The reversal of these parameters by EPA or EPA–HA implies its role in restoring subchondral bone mass and preserving trabecular connectivity. Compared with the sham group at 8 weeks, BV/TV was significantly decreased in the ACLT and HA groups, while the Tb.Pf values were significantly increased (Fig. 6f, g). Interestingly, this effect was diminished in the EPA–HA group, but not in the EPA group (Fig. 6f, g). We next performed CD44 staining in knee joint sections of mice at 4 and 8 weeks after ACLT surgery. Compared with the sham group at 4 and 8 weeks, the number of CD44-positive articular chondrocytes was significantly increased in the ACLT group (Fig. 6h). This effect was markedly diminished in the HA, EPA and EPA–HA groups at 4 weeks, and in the EPA and EPA–HA groups at 8 weeks (Fig. 6h).

## Discussion

This study is the first to show that, in addition to its anti-inflammatory properties, EPA can regulate F-actin organization and mechanical properties in IL-1α-treated porcine chondrocytes and human OA chondrocytes. It is well known that healthy chondrocytes exhibit a differentiated phenotype characterized by a rounded morphology, cortical F-actin organization and absence of stress fibers. By contrast, chondrocytes exposed to various conditions—such as serial passaging, pro-inflammatory signaling or OA—display a dedifferentiated phenotype, with a fibroblast-like shape, formation of stress fibers and significantly altered mechanical properties (for review, see ref. ^50^). In this study, we showed that IL-1α-treated porcine chondrocytes displayed significantly increased MMP3 expression, cell area, F-actin intensity and Young’s modulus, as well as decreased COLII expression and cell circularity. These finding align with multiple studies on the effect of pro-inflammatory signaling on chondrocytes isolated from various animal species^5,51–55^. Furthermore, we demonstrated that EPA treatment was able to mitigate IL-1α-induced changes in chondrocyte marker genes, F-actin organization and mechanical properties. Previous studies have reported that isolated human OA chondrocytes exhibited increased F-actin intensity and mechanical properties^56,57^. Here, we showed that EPA treatment decreased MMP3 expression, cell area, F-actin intensity and Young’s modulus, while increasing COLII expression and cell circularity in human OA chondrocytes. Taken together, these results demonstrate that EPA plays a role in restoring chondrocyte mechanics under OA conditions.

In OA, CD44 expression is increased in IL-1α-treated porcine chondrocytes, human OA chondrocytes^33^, mouse OA cartilage^58^ and human OA cartilage^43^. In addition, the level of CD44 expression in articular cartilage correlates with the severity of knee OA^59^. A recent study showed that ectopic expression of CD44 increased MMP3, MMP13 and COX-2 expression in mouse articular chondrocytes, while CD44 deficiency attenuated cartilage degeneration in an OA mouse model^58^. A recent single-cell analysis identified CD44 and JUN as candidate marker genes for hypertrophic chondrocyte and homeostatic chondrocyte in OA, respectively^60^. These results indicate that CD44 plays a critical role during OA pathogenesis. Consistent with previous studies, we found that CD44 expression was upregulated in IL-1α-treated porcine chondrocytes, IL-1α-treated porcine cartilage explants and mouse OA cartilage. We demonstrated that EPA treatment decreased CD44 expression in IL-1α-treated porcine chondrocytes, human OA chondrocytes, IL-1α-treated porcine cartilage explants, human OA cartilage explants and mouse OA cartilage. Consistent with our results, previous studies found that EPA treatment significantly suppressed CD44 expression and cell migration in colorectal cancer stem cells and breast cancer cells^61,62^. Thus, this study provides both in vitro and in vivo evidence demonstrating that EPA reduces CD44 expression in articular chondrocytes and cartilage under OA conditions.

Our data revealed a nuanced dual role of EPA in cartilage biology. While EPA ameliorated cartilage degradation and decreased expression of CD44 and p-p65 in IL-1α treated porcine cartilage explants, its standalone application paradoxically elicited mild pro-inflammatory effects in normal cartilage. This was indicated by elevated proteoglycan loss (Fig. 4d), increased OARSI scores (Fig. 4f) and elevated expression of CD44 and p-p65 (Fig. 4g–i). This paradoxical phenomenon aligns with the context-dependent metabolism of EPA. In the absence of inflammatory stimuli (for example, IL-1α), oxidized EPA derivatives such as PGE3 and LTB5—although substantially less potent than their arachidonic acid-derived counterparts (PGE2 and LTB4)—can weakly activate inflammatory pathway, triggering mild inflammation and matrix remodeling^63,64^. Conversely, under inflammatory conditions, these EPA metabolites competitively inhibit the arachidonic acid cascade, thereby suppressing the production of pro-inflammatory mediators. Notably, this mechanistic duality was corroborated in bovine cartilage explants, where EPA or DHA alone induced a mild catabolic response with increased sGAG loss^8^. From a clinical perspective, while EPA’s therapeutic benefits probably outweigh potential risks in inflammatory arthritis, co-formulation with antioxidants could mitigate its baseline pro-inflammatory activity. Future studies should focus on establishing dose–response thresholds and elucidating temporal dynamics in human OA models.

Although the regulation of CD44 expression in cancer cells has been well studied, how CD44 expression is regulated in chondrocytes remains unclear. In cancer cells, NF-κB and AP-1 regulate CD44 expression by binding to a conserved region located upstream of the CD44 promoter, and inhibition of NF-κB reduces CD44 expression and decreases cell proliferation and invasiveness^65,66^. In this study, we showed that EPA decreased CD44 expression in IL-1α-treated chondrocytes by inhibiting NF-κB p65 signaling activation but not AP-1 signaling. Moreover, we showed that EPA decreased the expression levels of CD44 and p-p65 in IL-1α-treated porcine chondrocytes, IL-1α-treated porcine cartilage explants and human OA cartilage. Taken together, these results provide both in vitro and in vivo evidence supporting the effect of EPA in reducing CD44 expression by inhibiting the NF-κB p65 signaling in articular chondrocytes and cartilage under OA conditions. KLF4 acts as a negative regulator of CD44 promoter activity by binding to the CD44 promoter region to repress its transcription and that of its variants, as indicated by the significant upregulation of CD44 expression following genetic ablation of KLF4 in pancreatic cancer cells derived from KLF4^flox/flox^ mice^67^. Interestingly, while KLF4 was upregulated by IL-1α, this response appeared insufficient to counteract the dominant pro-inflammatory effects of NF-κB on CD44 expression. This highlights the complexity of transcriptional networks in OA pathogenesis, where the balance between opposing regulators may determine disease progression. Future studies could explore whether KLF4 synergizes with EPA or fine-tunes CD44 dynamics under OA conditions. In addition to transcriptional regulation, CD44 activity is modulated by proteolytic cleavage into fragments (for example, CD44 extracellular truncation and CD44 intracellular domain) via metalloproteinase and γ-secretase activity, a process exacerbated in OA chondrocytes and inflammatory conditions^33^. Given that NF-κB activation promotes both CD44 transcription and metalloproteinase secretion, EPA’s inhibition of p65 phosphorylation may indirectly reduce CD44 fragmentation by limiting substrate availability and protease activity. Future studies are needed to test EPA’s direct effects on CD44 cleavage.

So far, no studies have investigated the effects of CD44 activation on chondrocyte mechanics. In this study, we showed that A6 induced CD44 activation in porcine chondrocytes, indicated by decreased cell circularity, increased F-actin intensity and increased Young’s modulus. This effect was inhibited by EPA treatment, demonstrating that EPA directly inhibited CD44-mediated changes in chondrocyte mechanics. CD44 activation is a complex and dynamic process regulated by various mechanisms, including HA molecular weight, CD44 isoforms, CD44 clustering, lipid rafts and posttranslational modifications (for review, see refs. ^68,69^). It is well established that EPA modifies lipid raft organization and results in altered cell function^70^. Therefore, EPA may inhibit CD44 activation induced by A6 or other CD44 ligands by modulating lipid raft organization. HA, the major ligand for CD44, is a nonsulfated glycosaminoglycan polymer consisting of repeating disaccharides. Depending on its molecular weight, HA exhibits various biological effects that play a pivotal role in cartilage ECM homeostasis. For example, LMWHA exhibits pro-inflammatory effects, and its distribution is associated with the risk of OA progression^71^, whereas HMWHA exhibits anti-inflammatory effects and its intraarticular injection is clinically used to treat early-stage OA. In this study, we showed that LMWHA increased the Young’s modulus of porcine chondrocytes, while HMWHA inhibited either LMWHA- or IL-1α-induced increase but alone did not affect the Young’s modulus. These results exhibited trends similar to those observed in rats, where HMWHA inhibited LMWHA-induced mechanical hyperalgesia, while HMWHA alone did not affect the nociceptive threshold^72,73^. Recently, HMWHA has been shown to serve as a pericellular coat that limits the mobility of CD44 and phagocytic receptors in macrophages^74^. Such a barrier on the cell surface could thus inhibit LMWHA- or IL-1α-induced CD44 activation and associated changes in chondrocyte mechanics. Taken together, these results demonstrate that CD44 activation plays an important role in regulating chondrocyte mechanics, and that EPA might restore the imbalance in chondrocyte mechanics caused by CD44 activation under OA conditions.

Intraarticular injection of HA is one of the most commonly used nonoperative treatments for early-stage OA. Although increasing evidence indicates that HMWHA exhibits better rheological properties, greater anti-inflammatory and antioxidative effects compared with LMWHA (for review, see ref. ^75^), its clinical efficacy in knee OA remains debated. One possible reason is that, in the pro-inflammatory microenvironment during OA development, HMWHAs are gradually degraded into LMWHAs and smaller fragments by hyaluronidases and reactive oxygen species, leading to increased CD44 expression and enhanced LMWHA–CD44 interactions, which impair chondrocyte mechanics and exacerbate inflammatory responses. In addition, previous studies have reported the use of EPA for intraarticular injections in mice, demonstrating its potential to reduce inflammatory responses^12^. However, the oily nature of EPA presents delivery challenges, as it may be difficult to achieve uniform dispersion within the synovial fluid, and its cellular uptake route remains uncertain. To address these challenges, this study utilizes a highly stable EPA emulsion, prepared by emulsifying EPA into a nano-emulsion using Food and Drug Administration-approved polymers. Functionally, this EPA emulsion-integrated HA injection restored chondrocyte mechanics and reduced CD44 expression while maintaining original HA’s performance in terms of rheological properties and other therapeutic effects. We found that, compared with HA, EPA emulsion or EPA–HA injection significantly ameliorated ACLT-induced cartilage degeneration and subchondral bone remodeling, and reduced CD44 expression in mice. Taken together, these results demonstrate that intraarticular injection of EPA emulsion or EPA–HA injection protects against ACLT-induced OA development in mice.

The present study had several limitations. First, our findings, while mechanistic, were generated in a controlled system that does not account for genetic, metabolic and environmental variability in patients with OA. Future studies incorporating patient-derived cells or stratified clinical cohorts will be essential to evaluate EPA’s efficacy across OA subtypes. Second, it is important to note that HA’s molecular weight distribution may influence EPA–HA’s stability and efficacy over time. It is likely that higher-molecular-weight HA prolongs joint retention but delays EPA release. Therefore, future studies should investigate how HA molecular weight modulates EPA’s therapeutic window, enabling personalized formulation strategies for patients with OA. Third, another limitation of this study is the lack of investigation on EPA–HA’s biodegradation kinetics and retention time within the joint microenvironment. Future studies using fluorescently labeled EPA–HA or in vivo imaging will be essential to optimize its delivery efficiency and therapeutic window. Fourth, it should be noted that our RNA-seq-based findings are limited to transcriptional changes under static culture conditions. Mechanosensitive pathways (for example, integrins, Piezo1 and primary cilia) may require mechanical stimulation or posttranslational activation, which should be explored in future studies using dynamic loading models and functional assays.

In summary, our in vitro tests indicated that EPA significantly decreased CD44 expression by inhibiting p65 phosphorylation and directly inhibited CD44 activation, thereby reducing CD44-mediated changes in chondrocyte mechanics (Fig. 6i). Our in vivo tests showed that EPA significantly alleviated articular cartilage degeneration and reduced CD44 expression in IL-1α-treated porcine cartilage, human osteoarthritic cartilage explants and OA mice. Importantly, EPA–HA injection protected against cartilage degeneration more effectively than HA in mice. This study identifies CD44 as a key factor in regulating chondrocyte mechanics in the pathogenesis of OA. This study suggests that EPA–HA injection might be a novel therapeutic approach for OA treatment.
